# Large-scale generation of differentiated cells to achieve regenerative medicine

**DOI:** 10.1186/scrt399

**Published:** 2014-01-20

**Authors:** Katsunori Sasaki

**Affiliations:** 1 Department of Histology and Embryology, Shinshu University School of Medicine, 3-1-1 Asahi, Matsumoto, Nagano 390-8621, Japan

## Abstract

The effects of microgravity and fluid dynamic stress on embryoid bodies generated from pluripotent stem cells induce and direct their differentiation. Using this hydrodynamic effect combined with exogenous factors and three-dimensional culture, a new technique has been developed to produce functional, effective, and safe hepatocytes for transplantation. The evolution of this technique will lead to automated production of a large number of differentiated cells and will significantly contribute to regenerative medicine.

## 

To translate regenerative medicine techniques using pluripotent stem cells (PSCs) from laboratories to the clinic, we have to overcome several limitations. The first step is development of simple, inexpensive, and mechanically automated methods to generate large numbers of differentiated cells from undifferentiated PSCs.

Zhang and colleagues proposed a new technique to generate a large number of functional hepatocytes [1]. They developed a three-dimensional culture system using a rotating bioreactor [2]. Hepatic differentiation is induced by microgravity, dexamethasone, dimethyl sulfoxide, hepatocyte growth factor, fibroblast growth factor-4, and insulin–transferrin–selenium at 1 × 10^6^ mouse embryonic stem cells (ESCs)/ml and a rotational speed of 25 rpm. In the first week of culture, typical embryoid bodies (EBs) appear and then differentiate into hepatocyte-like cells by 3 weeks of culture. This method increases the total number of cells by about 3 × 10^3^-fold. Compared with two-dimensional culture, the generated hepatocyte-like cells express hepatocyte marker genes and produce hepatocyte-associated proteins. Their functions were confirmed by albumin production, the activity of endogenous cytochrome P450 enzymes, low-density lipoprotein uptake, glycogen production, and indocyanine green staining. Day 14 EB-derived cells were injected into the spleens of nude mice. At 1 month post-transplantation, a large number of EB-derived cells were detected in recipient livers and no tumors were observed in recipient organs. Therefore, hepatocytes grown in this rotating bioreactor are safe and effective for use in transplantation.

The formation of EBs is the first step in conventional methods to differentiate PSCs [3]. EBs consist of the three germ layers and mimic the early period of embryogenesis. Three kinds of research regarding EBs should be taken up to refer to the background, significance, and perspective of the above research.

First, EB size is a parameter that affects differentiation. Bauwens and colleagues created micropatterned Matrigel islands on dishes using a printing method to regulate the size of human ESC colonies (200, 400, and 800 μm) [4]. Small colonies express higher levels of the *Gata* gene and lower levels of the *Pax6* gene, and exhibit endoderm characteristics. Large colonies show converse gene expression and a tendency for neural differentiation. These colonies were collected and resuspended in human ESC differentiation medium to form EBs for 4 days and were then transferred to plates for EB outgrowth. Among EBs generated from a high ratio of *Gata/Pax6-*positive colonies, larger EBs showed higher mesoderm and cardiac induction. Conversely, among EBs generated from a low ratio of *Gata6/Pax6*-positive colonies, smaller EBs exhibited higher cardiac induction. These results suggest that differentiation may be controlled by changing the EB size.

Second, the speed of the rotary culture regulates EB size. Carpenedo and colleagues demonstrated that rotary motion at 25 rpm generates the largest and fewest EBs from mouse ESCs, and 55 rpm yields the smallest EBs [5]. These generated EBs were uniform and expressed marker genes of the three germ layers. The EB size can therefore be controlled without the micropatterning technique. Moreover, an increase of endodermal gene expression and histological examination of cystic EB formation suggest that differentiation toward endoderm is accelerated in rotary-formed EBs.

Third, rotary culture involves fluid dynamics and microgravity that regulate EB differentiation. For example, ESCs exposed to fluid shear stress (5 dyn/cm^2^) show increased expression of endothelial marker genes, and EBs generated from these cells demonstrate the same tendency [6]. Rotary culture not only forms EBs and regulates their size, but also causes varied fluid dynamics and microgravity, and applies shear stress and gravitational effects on PSCs and EBs. Changing the fluid dynamics and microgravity may result in more selective differentiation.

Computational analysis combined with rotary culture by Sargent and colleagues revealed that different fluid dynamics occur in rotating dishes at 25, 40, and 55 rpm [7]. This finding suggests that shear stress modulates the EB structure including the cellular organization and morphology of the spheroids. Compared with static culture, rotary culture increases the expression of cardiomyocyte marker genes. Mogi and colleagues have shown that 80% of EBs created at rapid speed (100 rpm) beat after outgrowth without the addition of growth factors [8]. However, at 120 rpm the number of beating EBs decreases by one-half. Computational fluid dynamic analysis showed abrupt changes of the fluid dynamic phase from 100 to 120 rpm. Zhang and colleagues found that selective differentiation by microgravity is able to generate cells with endodermal phenotypes from PSCs [1].

Microgravity-associated methods may realize selective differentiation of PSCs to supply large quantities of differentiated cells and satisfy the demand of regenerative medicine. These methods may not be fully developed, suitable equipment has not been established, and the optimal conditions have not been determined. However, such limitations may be overcome by investigating techniques such as three-dimensional culture systems and the addition of exogenous factors as suggested by Zhang and colleagues [1].

## Abbreviations

EB: Embryoid body; ESC: Embryonic stem cell; PSC: Pluripotent stem cell.

## Competing interests

The author declares that he has no competing interests.
